# Impact of the COVID-19 pandemic on accredited conformity assessment bodies: insights from a multinational study

**DOI:** 10.1007/s00769-022-01514-x

**Published:** 2022-08-29

**Authors:** Claudia Koch, Parsa Asna Ashari, Mona Mirtsch, Knut Blind, Pavel Castka

**Affiliations:** 1 S.2 Accreditation and Conformity Assessment, Bundesanstalt für Materialforschung und -prüfung (BAM), Rudower Chaussee 11, 12489 Berlin, Germany; 2 Technische Universität Berlin, Berlin, Germany; 3 Fraunhofer Institute for Systems and Innovation Research, Karlsruhe, Germany; 4 University of Canterbury, Christchurch, New Zealand

**Keywords:** COVID-19, Conformity assessment, Quality infrastructure, Accreditation, Laboratory

## Abstract

The COVID-19 pandemic posed new and manifold challenges to organizations and their operations worldwide. Conformity assessment bodies (CABs), such as testing or medical laboratories, certification, and inspection bodies, were also affected by the associated disruptions. Their role in this crisis is highly relevant, as CABs are essential pillars of the quality infrastructure: their activities ensure that products and services meet requirements as defined in standards and regulations, thereby contributing to their safety and reliability. The question arises of how CABs and their operations were affected by the pandemic and how they responded. To this end, we present the results of an international survey of 986 CABs of all types in Germany, the UK, Italy, and New Zealand. Overall, CABs reported, on average, a reduction in demand for their services during the pandemic, facing restrictions in all countries. In addition, the pandemic had an overall negative impact on the CABs’ investment and innovation activities. However, investments in digital infrastructure were increased as a countermeasure, with CABs reporting a higher need for digitalization. The paper highlights and discusses results from in-depth analyses relevant to policymakers and industry alike.

## Introduction

Conformity assessment (CA), such as testing, certification, and inspection, demonstrates whether specified requirements, as stated, e.g., in standards, are fulfilled [1]. With that, CA is central to the effectiveness of standards to ensure their intended use and, consequently, their impact. The COVID-19 pandemic has posed new and manifold challenges to organizations and their operations worldwide, such as travel restrictions, remote operations, and unpredictable demand [2–4]. Conformity assessment bodies (CABs) were also affected by these challenges and associated disruptions [5–8].

CABs are essential pillars of the quality infrastructure: their services contribute to the safety and reliability of products and services [9] and play a fundamental role in innovation [10]. They are a cornerstone of trade by establishing trust and transparency [11–15]. CABs essentially provide a critical link between regulators, industry, and markets [16]. Considering CABs’ roles and functions in the economy, the question arises of *how CABs were affected by the pandemic and how they responded to it*. As the pandemic continued to challenge the supply chains, CABs needed to find ways to continue delivering their services. Anecdotal evidence suggests that the provision of CA services has been challenging due to staff shortages, travel restrictions, and a lack of competence to operate remotely [17, 18]. In some countries, CABs were not classified as ‘essential services’, which made the provision of their services even more challenging. However, critical sectors of the economy (i.e., food manufacturing and supply, hospitals, and medical testing) relied on CABs. Likewise, new products (i.e., ventilators) and facilities that switched their production lines to products such as masks or hand sanitizers needed CABs to provide licenses to operate [19].

Given the importance of CA to the global economy, it is vital to understand the issues that CABs faced during the crisis and their vulnerability, as well as to identify potential support measures. To support this aim, we discuss the results of an international survey of 986 CABs carried out in 2020 covering testing and medical laboratories, certification, calibration and inspection bodies, and others, i.e., validation and verification, proficiency testing, and production of reference material. The study considers only accredited CABs. We refer exclusively to CABs that have the competence to carry out conformity assessment services approved by a national accreditation body—against the specifications in ISO/IEC standards which oftentimes also require risk analysis to be in place [1]. The results discuss the impact of the pandemic in terms of economic and operational issues. The study uses survey data from four countries—Germany, the UK, Italy, and New Zealand—to account for differences in policy responses and economic disruptions in different countries. While they are all industrialized countries that allow for comparison in general terms, they differ in terms of the time, way, and extent to which the pandemic hit them. With this international comparative study, we not only address the actual economic situation of CABs in the pandemic but also investigate how it impacts their digitalization.

So far, however, there has been no comprehensive study that empirically determines the impact of the COVID-19 pandemic on the different types of CABs worldwide. We fill this gap by investigating the short-term impact of the pandemic on CABs and analyzing the ability of CABs to deliver their services to industry and society and fulfill their role in the economic system. Further, we investigate to what extent the pandemic has impacted the digitalization of CABs (and vice versa). We contribute to the emerging literature on the impact of the COVID-19 pandemic on various sectors, shedding light on the situation of a sector whose customers depend on CA services in these challenging times more than ever and that heavily relies on accreditation as an “additional layer of trust”. Unlike previous studies that draw from anecdotal evidence or limited samples, we draw from a large sample of CABs that operate across the globe.

## CABs in the pandemic

### Conformity assessment

**Fig. 1:**
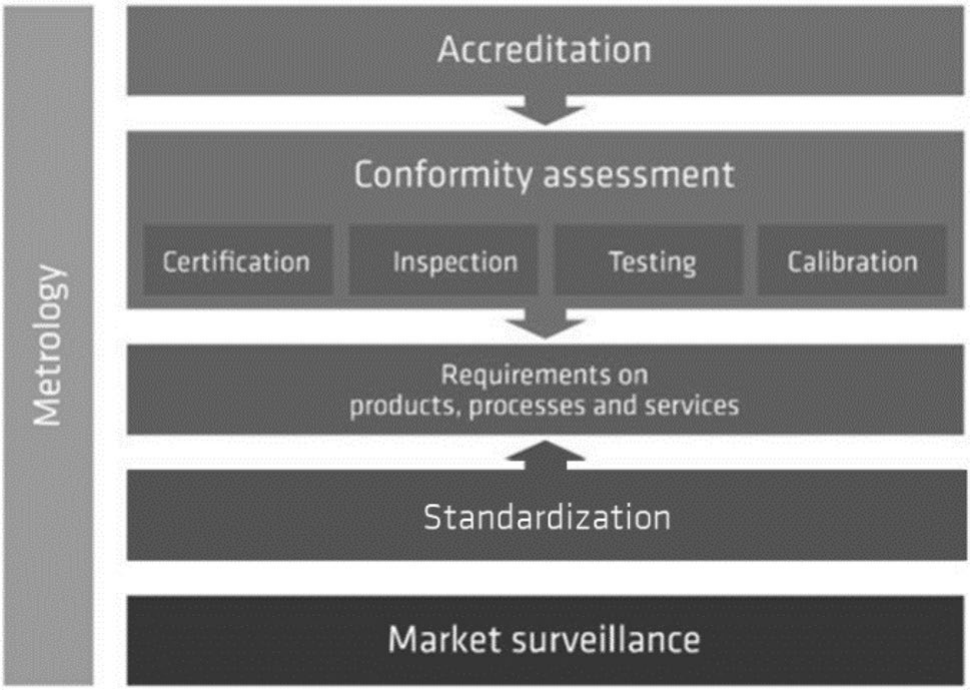
Quality infrastructure. *Source*: Koch and Blind [28]

CA is a central pillar of the national quality infrastructure (see Fig. 1), that is, the “system comprising the organizations (public and private), together with the policies, relevant legal and regulatory framework, and practices needed to support and enhance the quality, safety and environmental soundness of goods, services and processes” [20]. CA helps transparently and reliably differentiate goods and services as to whether and how they conform to requirements set out in standards or regulations, thus increasing the importance of such standards [10]. The benefits of standards and standardization in terms of economic efficiency and market access can only be achieved by demonstrating such conformity [21].

CA includes activities such as testing, inspection, validation, verification, certification, and accreditation [1]. Harmonized requirements for the corresponding practices and operations of CABs, bodies that perform CA activities, are set out in international standards published by ISO (International Organization for Standardization) and IEC (International Electrotechnical Commission). Against these standards, CABs can seek accreditation to attest their competence, impartiality, and consistent operation in performing specific CA activities. Standardized CA practices are vital to domestic and international trade and to safeguarding public goods such as the protection of health, safety, and the environment [21]. Internationally harmonized and recognized standards and CA practices, among others, are vital to avoid unnecessary and burdensome double-testing in cross-border trade [22]. In previous studies, accredited CABs have been associated with positive impacts on the gross domestic product [23–25]. Blind et al. [11] also found significant positive effects of standards and certifications on exports, which are enhanced by harmonized procedures and corresponding multilateral recognition agreements. The corresponding harmonized CA standards indeed help overcome trade barriers [21]. Besides these trade-enhancing effects, CAs in the form of third-party certifications have also been found to increase product safety [26].

CABs may seek accreditation to provide their services within predefined scopes. The role of accreditation (as an activity of CA) is to independently confirm the competence of CABs to execute the conformity assessment-related tasks. In addition, the aim is to build trust and enable the international acceptance of CAs through mutual recognition agreements [1]. In the Member States of the European Union, accreditation “is part of an overall system, including conformity assessment and market surveillance, designed to assess and ensure conformity with applicable requirements. The particular value of accreditation lies in providing an authoritative statement of the technical competence of bodies whose task is to ensure conformity with the applicable requirements” (EU Regulation 765/2008) [27]. To be accredited, CABs provide services in accordance with applicable standards, such as those published by ISO and IEC with ISO/IEC 17000:2020 [1], which “specifies general terms and definitions relating to conformity assessment (including the accreditation of conformity assessment bodies) and to the use of conformity assessment to facilitate trade.”

Industry relies heavily on CA, especially in its increasingly international supply chains. Here, CA ensures transparency and trust and demonstrates the fulfillment of requirements for products and services. CABs are, therefore, an indispensable cornerstone of our economies. However, the established, interdependent network was severely disrupted by the outbreak of the COVID-19 pandemic in 2020.

### Conformity assessment during the pandemic

COVID-19 was first reported in Wuhan, China, in late 2019 and has since evolved into a worldwide pandemic with almost 487 million confirmed cases and more than 6 million deaths as of March 2022 [29]. The outbreak of the coronavirus SARS‐CoV‐2 led to an unprecedented global health, social, and economic crisis. The latter was fueled by deep uncertainty as well as travel and other restrictions imposed by governments worldwide to contain the spread of the virus [30].

Global supply chains were heavily disrupted, manifesting in “ripple effects”, with supply chain components affected sequentially or concurrently. The pandemic led to the closure of production and distribution activities, downstream interruptions, and spillovers in key sectors of the global economy. As a result, international goods trade was vastly reduced, and demand and supply shocks occurred, ultimately causing worldwide economic disruptions and recessions [31, 32]. Therefore, in addition to human control and public health measures, policy responses included monetary and fiscal measures to cope with the impact of the COVID-19 pandemic [32].

With industries stagnating worldwide, CABs, as a central pillar of (global) trade and economic activity, experienced a decline in demand for their services with the onset of the pandemic [33]. On the other hand, CA services remain fundamental to the functioning of the economy. The continued supply of their services has been vital for the maintenance and recovery of value chains, for evaluating products and services—even more so for areas that have been essential for coping with the crisis [33]. CABs have had to respond quickly to the increasing demand for specific services, e.g., to ensure the supply of medical devices and pharmaceuticals or the delivery of protective equipment. In particular, medical laboratories play a crucial role in diagnostics and the development of vaccines and treatments needed to overcome the pandemic [34]. However, the ability of CABs to provide such services has been constrained, e.g., due to staff or materials shortages or travel bans [5].

While the impact of the pandemic on CABs varies across the sectors and markets they serve, the type of CA they offer, the stage of the value chain affected, or the importance of the activity, the recovery of the CA sector depends on the recovery of the underlying value chains [18]. The pandemic demonstrated the importance of the ability of organizations to master crises and cope with the challenges associated with the pandemic, especially the restrictions imposed to contain the spread of the virus and the disruptions to global value chains. At the same time, opportunities emerged for the sector, with CABs providing essential services that are key to fighting the pandemic. While some CABs faced severe threats to their economic viability, others were able to respond and adapt, e.g., by implementing new digital solutions [7, 18].

As core activities of CABs, on-site audits and inspections were heavily affected by restrictions as countermeasures to the pandemic, especially in the early phase, forcing them to adapt quickly [7]. Castka et al. [6] found a significant increase in remote auditing and the use of information and communication technologies among certification bodies involved in voluntary sustainability standards in response to the pandemic, a development that has already shaped a “new normal”. While pre-pandemic adoption of remote auditing in this area was slow, the authors find that the pandemic has accelerated the adoption of digital technologies in such CABs, triggered by travel bans and restrictions on access to customer sites.

## Research method and context

### Research approach

To get an overview of the situation of CABs during the pandemic, we set up an online questionnaire covering important aspects of the provision of CA services, broadly economic and operational impact (e.g., demand for their services or constraints on their operations), resources and preparedness, and digitalization of CA services. Questions were designed with either yes/no responses or five-point Likert scales, e.g., with 1 for "not important" and 5 for "very important", or −2 for “strong decrease” and +2 for “strong increase,” respectively. We also included multiple-choice questions. The questionnaire was designed to account for the differences between the various types of CABs, such as testing laboratories or certification bodies, through various filter questions. In total, the questionnaire comprised up to 38 questions.

### Data collection and sample

The data were collected as part of the QI-FoKuS (“Quality Infrastructure—Research for Conformity Assessment and Safety”) initiative (www.qi-fokus.de). Conducted by the Bundesanstalt für Materialforschung und -prüfung (BAM) and Technische Universität Berlin in collaboration with the Fraunhofer Institute for Systems and Innovation Research, and the University of Canterbury, QI-FoKuS aims to create a data basis to better understand the benefits of CA and accreditation and identify future trends.

Therefore, we contacted all accredited CABs in Germany listed in the official register of the German accreditation body DAkkS (a total of 3204 CABs) directly via email in June 2020. In Italy, the UK, and New Zealand, we cooperated with the local accreditation bodies as multipliers, namely Accredia (Italy), UKAS (UK), and IANZ & JAS-ANZ (New Zealand), which sent invitations to our survey to their accredited CABs between July and November 2020. In total, we received 986 valid responses from Germany (555), Italy (240), the UK (71), and New Zealand (120).

In our analysis, we differentiate (in-line with relevant ISO standards) according to the ISO/IEC 17000 series between testing and calibration laboratories (ISO/IEC 17025) [35], medical laboratories (ISO 15189) [36], and certification bodies for products, management systems, and persons (ISO/IEC 17065, ISO/IEC 17021-1, ISO/IEC 17024) [37–39]. The other CA activities such as inspection (ISO/IEC 17020) [40], validation and verification (ISO/IEC 17029) [41], proficiency testing (ISO/IEC 17043) [42], and production of reference materials (ISO 17034) [43] are grouped as “other.”

**Fig. 2:**
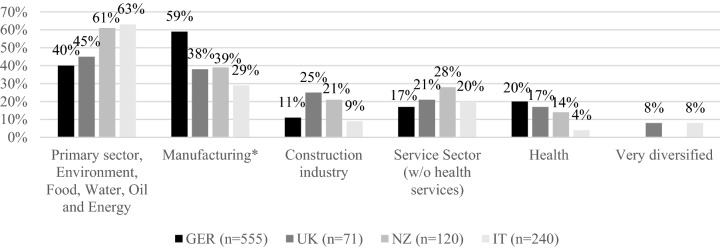
Sectors served by participating CABs in the national samples compared (% of CABs that engage in aggregated sectors). *Note*: Multiple answers are possible with up to three sectors per CAB. *includes Mechanical and plant engineering; Electrical engineering; Metal industry; Chemicals, rubber, plastics, pharmacy; Textiles; Vehicle construction

In our sample, some of the responding organizations offer more than one service (e.g., operating a testing laboratory and a product certification body). Two-thirds of all CABs in our sample are testing laboratories, followed by certification bodies (19 %). One in five respondents is an internal CAB, i.e., one that belongs to a larger corporation such as a manufacturing company for which it provides its services internally. Most CABs in our sample are small and medium-sized enterprises with fewer than 250 employees (ranging from 61 % in the UK to 90 % in Italy). As shown in Fig. 2, the main sectors served by the CABs vary across the four countries: the main customers of German CABs are from the manufacturing sector, while the primary sector/food/water/energy was dominant in the other countries. The international orientation of the CABs varies widely as well: in all countries except Italy, the majority of CABs engage in international activities (having international customers and/or being active in other countries). German CABs are leading in this respect, with 67 % being internationally active, while in Italy, 74 % of the surveyed CABs focus on domestic markets. Table 1 summarizes the data describing the sample.

**Table 1:** Sample description. Note: N refers to the surveyed CABs, not the sum of offered CA activities.

	All *N* = 986	GER *N* = 555	UK *N* = 71	IT *N* = 240	NZL *N* = 120
*CAB activity*					
Testing laboratory	68 %	63 %	66 %	75 %	74 %
Certification body	19 %	22 %	18 %	15 %	13 %
Calibration body	18 %	23 %	13 %	10 %	17 %
Inspection body	12 %	10 %	17 %	13 %	18 %
Medical laboratory	5 %	6 %	10 %	0 %	2 %
All other	3 %	3 %	3 %	4 %	3 %
*Company size*					
<10 employees	42 %	37 %	27 %	53 %	48 %
10-49 employees	36 %	38 %	34 %	37 %	26 %
50-250 employees	14 %	16 %	21 %	8 %	14 %
Over 250 employees	8 %	8 %	15 %	3 %	8 %
I don’t know	1 %	0 %	3 %	0 %	3 %
*International activities*					
Yes	54 %	67 %	55 %	26 %	47 %
No	46 %	33 %	45 %	74 %	53 %
*Operational focus*					
Primarily CA for external customers	36 %	39 %	25 %	35 %	27 %
CA for external customers one activity among others	35 %	36 %	51 %	24 %	38 %
Primarily CA within an organization	21 %	15 %	18 %	34 %	28 %
Other	9 %	10 %	6 %	8 %	7 %

### Analysis

For our empirical analysis, we present descriptive statistics for each country and analyze them comparatively. Significance tests were performed using QResearch software [44]. To test for significant differences (e.g., between countries or CA activities), we conducted Pearson’s Chi-square tests of independence with false discovery rate corrections [45]. When results differed significantly between countries or due to specific characteristics of the CABs, we reported these findings, documenting the corresponding *p* value.^1^

### Epidemiological situation in the four survey countries

The composition of the sample in terms of company size and other organizational characteristics is not the only aspect to be considered when assessing the survey results. The pandemic hit the four countries at different times and with different intensities. Accordingly, governmental responses and economic and societal impacts also varied. Because the start of the survey differed among the four countries due to their dependence on national cooperation partners, the prevailing circumstances and epidemiological situations must be considered when comparing the results.

COVID-19 cases started to rise in some regions of Italy in February 2020. The virus then spread to Germany and the UK in mid-March with a similar pattern of case development, with cases peaking in late March or April 2020, declining during the summer months, and rising again in September 2020 in a second wave. On the other hand, New Zealand showed a different pattern: after case numbers spiked in April, contagion rates remained relatively low. Overall, Italy and the UK were hit the hardest in terms of contagion rates and death tolls, while New Zealand had by far the lowest numbers [29, 46]. The epidemiological situation at the time of the national survey is displayed in Table 2. It shows that cases increased drastically again when the Italian survey started in October 2020, whereas the incidence in Germany and the UK was lower at their respective starting point (June 2020 in Germany, July 2020 in the UK), and restrictions were alleviated. The New Zealand survey, in contrast, started in October 2020, right after restrictions were renewed in some areas of the country.

**Table 2:** Epidemiological situation at the start of the survey. *Source*: ECDC (2021), own calculations.

Country (start of the survey)	14-day incidence of cases (per 100k inhabitants)	Cumulative number of cases (from 01/01/20) (per 100k inhabitants)	Cumulative number of COVID-related deaths (per 100k inhabitants)	Anti-COVID measures during the survey
GER (June 4, 2020)	7.2	220	10	Most restrictions eased
UK (July 21, 2020)	12.7	445	60	Most restrictions eased
NZL (Oct 6, 2020)	0.8	30	1	Most restrictions eased after restrictions had been reintroduced
ITA (Oct 12, 2020)	74.7	589	62	Some restrictions reintroduced

Governmental responses were most stringent in Italy and New Zealand, which imposed nationwide lockdowns that entailed the closure of all non-essential businesses, while in Germany, only retail, hospitality, and services involving human contact were shut down [47]. The last column of Table 2 summarizes governmental responses to the pandemic during the survey, while the first column displays the start date of each national survey.

## Results and discussion

### Economic impact of the pandemic

#### Order development

**Fig. 3:**
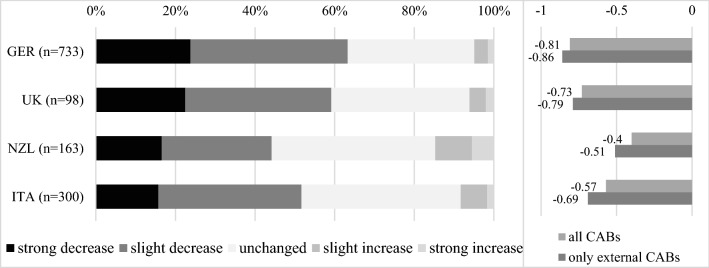
Impact of the pandemic on demand for CA compared to the pre-COVID-19 period, from −2 (strong decrease) to +2 (strong increase), distribution (left) and mean values (right) across countries. n=number of different fields of activities (e.g., testing laboratories, product certification bodies, etc.). *Note*: CABs can offer more than one activity.

Participants were asked to compare the *current demand for their services* (at the time of the survey) with the pre-pandemic period. On average, CABs reported a clear negative development (Fig. 3), both domestically and internationally. The smallest average decrease is experienced in New Zealand, where *less* than 50 % of laboratories and bodies reported a decline in demand for their services and 15 % even reported an increase in demand, and in Italy, as opposed to the largest decrease in Germany. As already mentioned (see 3.4), these country differences might also be caused by the survey timing and the associated restrictions.

Moreover, our sample shows that German CABs tend to have more customers from the manufacturing sector, which was severely disrupted at the beginning of the pandemic [48, 49] and whose global value chains were under pressure. In addition, the German economy is comparatively more integrated into international supply chains [50]. Therefore, this contributes to the German CABs being generally more severely affected. Overall, inspection bodies that rely on traveling to customers and performing on-site services were the hardest hit. On the other hand, medical laboratories were less negatively affected due to the indispensability of their services and reporting the comparatively most gains in orders. These gains can be attributed to new services in demand at the outset of the pandemic, such as PCR tests. The decline in demand in the four countries varied not only in magnitude but also in terms of the underlying reasons: while the Italian CABs attributed about 60 % of the decline to postponements to a later point in time, these figures were lower in the other countries, where a decline in new incoming orders was the most severe factor (about 50 % to 60 %). In the UK and New Zealand, the comparatively most CABs in the sample reported cancellations (significantly high share of 17 %, *p* = 0.02, and 20 %, *p* < 0.01).

Internal CABs (providing services to a company associated with itself) were generally less affected by the pandemic than external CABs (serving customers outside their own organization), as shown by the mean values in Fig. 3.

**Fig. 4:**
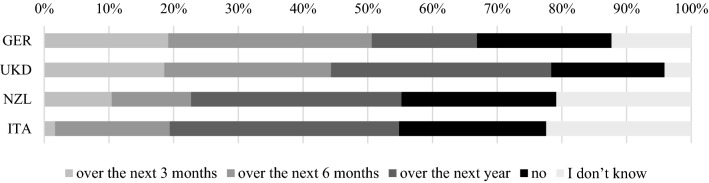
CABs’ expectations of recovery from decreasing demand.

While demand declined, CABs’ *customer base* was largely unaffected by the pandemic. However, 20 % of CABs in New Zealand and 19 % of medical laboratories across all countries were able to gain new customers, while almost one in three certification bodies for persons and management systems as well as 28 % of the UK’s CABs contended to have lost customers. The CABs were also asked whether and when they *expected the demand situation to recover* from a potential future normalization of overall economic activity. Distinguishing CABs by their activities, we find medical laboratories to be more optimistic and inspection bodies to be more hesitant. Overall, a clear difference between the countries was observed (Fig. 4): those countries surveyed earlier (Germany and the UK) were more optimistic about recovery, while respondents from New Zealand and Italy, surveyed several weeks later in October 2020 and experiencing the consolidating negative epidemiological and economic situation and associated insecurity, were less optimistic in their outlook. While half of the CABs in the UK and Germany (surveyed in June and July), where the pandemic had just passed its (first) peak, expected a recovery within the following six months, this was the case for only about 20 % in Italy and New Zealand. There, one in four fields of activity expected no foreseeable recovery at all, highlighting fading hopes for a quick end to the COVID-19 emergency in light of ongoing outbreaks and restrictions across the world.

Studies investigating various *industries served* by CABs show that supply chain disruptions during the pandemic pushed the manufacturing sector towards more robust local supply networks and digital technologies. This suggests that a sustained recovery of the sector from the crisis will require the likely, inertial restoration of previous supply chains [51]. Nevertheless, the manufacturing sector’s heavy dependence on commodities is an obstacle to companies’ operations, especially for less resilient small and medium-sized enterprises that suffer from the unpredictable duration of the crisis [52]. Manufacturers of non-essential products and services additionally face a decline in consumer demand due to their partial shutdown [53]. Other industries, such as the construction industry [54], the food and agricultural sector (despite its supply-chain nature and the delivery of essential products) [55], and the water supply sector [56] were found to be less affected, indicating a more inelastic sectoral demand. However, the energy industry faces a decline in electricity demand, productivity, and revenues [57, 58]. Overall, the development in the served industries during the COVID-19 pandemic, as well as the offered CA activities, determine how CABs suffered or benefited from the crisis.

#### Operational restrictions and government support

**Fig. 5:**
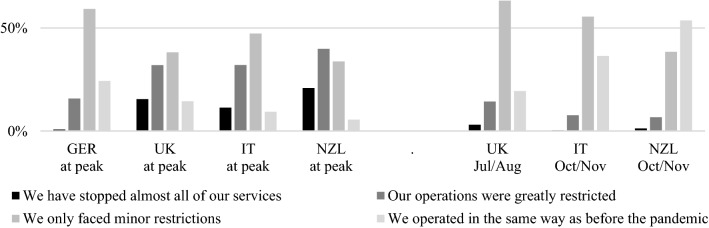
Restrictions faced at the first peak of the pandemic (March to June 2020) and at the time of the survey in the respective countries.

Governments imposed different measures, at different times and with different intensities, during the pandemic in the four countries, leading to restrictions on CABs’ operations. While the governments of Italy and New Zealand reimposed some restrictions, measures in Germany mainly were less extensive at the time of the survey (see Table 2). This is reflected in the restrictions that CABs faced and how these affected their operations. Therefore, we asked them how severely their *provision of services was affected*, both at the first peak of the pandemic in March/April 2020 and at the time of the survey (except for the German participants, who were surveyed as early as June 2020, very close to the peak). Given the lower governmental restrictions in Germany, most CA activities were reported to have faced only minor restrictions in their operations, and almost no CAB had to stop (almost) all services (see Fig. 5). In contrast, one in five CA activities in New Zealand was forced to cease (almost) all operations, and these figures are also comparatively high in the UK and Italy (15 % and 11 %, respectively).

Limited access to customers and travel restrictions were the most prevalent constraints faced by CABs in all countries. Employee absence was also an issue for 40 % of CABs experiencing restrictions, significantly more in the UK (63 %, *p *< 0.01). Bottlenecks in supply were experienced by 37 % of restricted CABs, primarily for consumables, protective equipment, and technical services. In New Zealand, 18 % suffered from bottlenecks in the supply of equipment and spare parts, compared to 8 % of CABs overall (*p *< 0.01).

However, during 2020, as restrictions were gradually relaxed in countries, many CABs experienced an easing of their operational situation, if not a return to normality. Thus, the collected data covered restrictions at the time of the survey, which in the case of the UK, Italy, and New Zealand was several months after the initial peak. At the time of the survey, less than 15 % of CA activities in Italy, the UK, and New Zealand were still heavily restricted. Relief was particularly prevalent in New Zealand, where 54 % of activities could be performed as before the pandemic. While most constraints were alleviated at that time, restricted access to customers remained an issue for 45 % of constrained CABs, while supply bottlenecks were still common in New Zealand.

The operational restrictions and the declining demand for their services meant that 2 % of all CABs saw their *business viability* under immediate threat (in Italy, as much as 5 %). However, 69 % of respondents claimed the survival of their business not to be at risk, while 29 % saw a risk of failure if no fundamental changes occurred in the six months following the survey. The threat to business viability is even more prevalent for external CABs: only 59 % contended not to be at risk, while 6 % saw an immediate threat.

In all four countries, governments responded to the pandemic by introducing financial *support measures* for companies suffering from the economic impacts of the pandemic, e.g., wage subsidies, loans, other subsidies, and tax credits. 45 % of all CABs in our sample reported having applied for government support measures, with Germany being the least likely to have done so (35 %, *p *< 0.01). Considering the longer-lasting negative impacts at the time of the survey in the other three countries, we found that CABs from there more frequently applied for such government support measures, most frequently in the UK. The share is considerably higher if we exclude the less affected internal CABs, thus yielding that 71 % of CABs in the UK, 60 % in New Zealand, and 66 % in Italy had sought government assistance. Not all types of CABs were equally affected: the number of medical laboratories under threat was significantly lower, yet one in four was affected (27 %, *p *= 0.01).

The overall provision of government support in Germany reveals that most aid is available to large enterprises (EUR 500 bn), followed by small (EUR 50 bn) and medium-sized enterprises (EUR 25 bn). Small enterprises, however, have applied for most of the available funds, indicating their urgent need for financial support to manage the crisis [59]. This is mainly in line with the findings from our survey: especially the smaller, external CABs, which suffered disproportionally from the external decline in demand, requested more governmental help than large or internal CABs that are part of large corporations.

Comparing the proportion of CABs applying for government help identified in our survey with the overall share of businesses explored in other studies reveals that the CA sector has a comparably lower propensity to seek financial aid—especially in Germany (see Table 3). However, this finding could be explained by several reasons that need further investigation. First, it might result from the CA sector being less affected by the COVID-19 pandemic. This indicates that CA, and thus the CA sector, is of fundamental importance to large parts of the economy and public goods such as health protection so that its services are still in demand at a rate disproportionate to the economy’s average. Second, accounting for their role in value chains, CABs may be affected only by a time lag, highlighting the need to reassess them at a later point in time.

**Table 3:** Application for governmental help from the CA sector and the entire economy. *Sources*: Own survey and other surveys.

	CAB sector total	CAB sector w/o internal CABs	Total economy
GER	35 %***	36 %	70 % (06/2020) [60]
UK	62 %	71 %	81 % (07/2020) [61]
ITA	59 %	66 %	70 % (04/2020) [62]
NZL	54 %	60 %	80 % (04/2020) [63]

***p < 0.01, **p < 0.05, *p < 0.1

Finally—given the vital role CABs play in the functioning of the economy for health and safety—we wanted to know whether they would be able to *meet their customers’ demand as the economy gradually recovers* from the shock and disruptions of the initial peak of the pandemic. 65 % of CABs were confident of meeting the demand, whereas only 2 % expected considerable delays. Again, the UK (*p *= 0.04) and New Zealand (*p *= 0.03), the last to be surveyed, were already more optimistic, with three out of four CABs claiming their ability to meet growing demand without delays. Almost 40 % of CABs expected some or severe delays in Germany, and more than 30 % of Italian CABs (surveyed late) also expected delays. The most common reasons for such expected delays were accumulated order backlog (54 %; 63 % in Germany, *p *< 0.01), sustained lack of employee availability (43 %), and persistence of travel restrictions (39 %).

#### Investments and innovation activities

Against this background, the study investigated the impact of the pandemic on the investment and innovation activities of CABs. The aim of the study was, first, to find out whether the pandemic had led to CABs changing their fields of activity, i.e., whether they had adapted to the new challenges and opportunities in their services. This was more often the case in those countries surveyed later than in the German CABs, which were surveyed as early as June 2020, only three months after the pandemic started. Overall, 19 % of CA activities expanded the *portfolio of services offered* and/or strengthened some activities—while this was the case for only 15 % in Germany. However, the vast majority did not adapt.

**Fig. 6:**
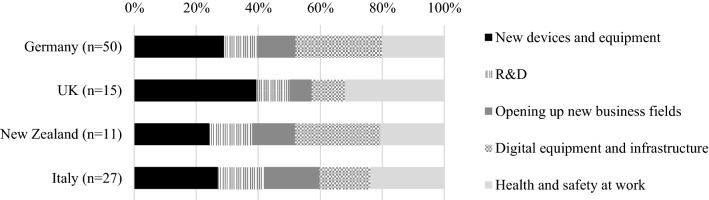
Areas where surveyed CABs directed additional pandemic-related investments. *Note*: n refers to the CABs, not their offered CA activities.

The pandemic impacted the investments of the CABs (Fig. 6): 43 % of the laboratories and bodies surveyed *postponed planned investments*, while only 9 % increased them. Again, we found differences at the country level: postponements ranged from only 31 % of CABs in New Zealand to 47 % of Italian CABs. Medical laboratories invested significantly more often (45 %, *p *< 0.01), while calibration and testing laboratories tended to defer investments more often (*p *< 0.01). The investments were especially allocated to new devices and equipment and necessary health and safety measures. In Germany and New Zealand, investments in digital infrastructure were made more frequently. The following section explores the role of digitalization in more detail.

### Digitalization

Digitalization in the context of the pandemic is analyzed from two perspectives: we shed light on whether and how digital tools and capabilities helped to master the crisis, and we take a closer look at how the pandemic affected the digital transformation of the CABs.

**Fig. 7:**
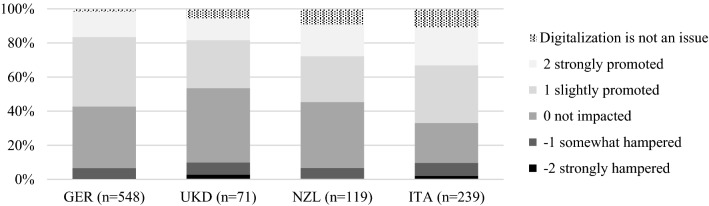
Impact of the COVID-19 pandemic on CABs’ digitalization processes compared. *Note*: n refers to the CABs, not their offered CA activities.

Given the constraints associated with the pandemic, 70 % of laboratories and bodies reported an increased *need for digitalization*, especially in Germany and Italy. Previous research has highlighted the pressure for companies to adjust their internal capabilities and resources to adapt to the challenges associated with the crisis and the accordant instability and uncertainty [64], including and especially in terms of digital transformation [65]. Given the reported needs, the pandemic has driven digitalization in German and Italian CABs significantly more than in the other countries (52 % on average; UK: 41 %, *p *= 0.03; NZ: 45 %, *p *= 0.06; see Fig. 7).

**Fig. 8:**
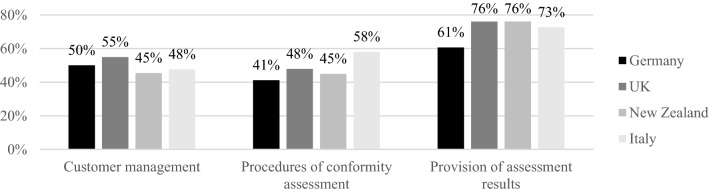
State of digitalization in core operational areas of CABs (% of CABs that claim to be “completely digitalized “or “predominantly digitalized” in these areas).

**Fig. 9:**
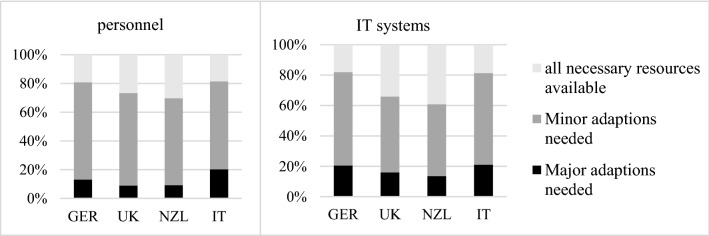
Availability of personnel and IT infrastructure according to the current need for digitalization.

This corresponds to the *state of digitalization at the beginning of the pandemic*: respondents from the UK and New Zealand were already more digitalized in major operational areas such as the provision of assessment results or the actual procedures of CA (with Italy leading the way here), and thus experienced less pressure to adapt (see Fig. 8). The assertion is supported by the availability of IT resources mentioned above but also by respondents’ assessments of the *need for further adaption* of IT and personnel to digital environments (or a lack thereof): 39 % of CABs from New Zealand (*p *< 0.01) and 30 % from the UK (*p *= 0.1) claimed their IT infrastructure was fully prepared for increasingly digital environments, while the share for Germany was significantly lower (16 %, *p *= 0.01). 20 % of German and Italian CABs saw major adaptions as necessary. CABs in New Zealand were also better prepared in terms of personnel, with 32 % (*p *= 0.04) being fully adapted to digitalization needs, while the share was significantly lower in Germany (18 %, *p *= 0.05). 15 % of CABs still felt the need for major adaptions in this area, significantly more for Italian CABs (21 %, *p *= 0.06) (see Fig. 9).

Digital technologies not only help organizations to better cope with the challenges of a crisis but also to seize opportunities [65]. Since the on-site delivery of their services was severely affected by the pandemic [6], the surveyed certification and inspection bodies were additionally asked whether they provided remote audits and inspections.

In line with previous findings [6, 7], *remote activities* were triggered: 70% of certification and inspection bodies reported doing so, with as many as 50 % of them doing so for the first time due to the pandemic. In Italy, the share of CABs providing remote audits and inspections was significantly lower, with 44 % refraining from doing so (*p *= 0.01). The most common reasons for CABs not to engage in remote audits and inspections were that they were not possible in terms of content (53 %), not permitted by laws and standards (50 %), provided limited insights (26 %), or not demanded by customers (22 %).

As crises bring not only challenges but also opportunities [66], the pandemic seems to have triggered an accelerated digital transformation in CABs, a development that is in line with other studies in other industries and countries as well [6, 65, 67–69], and which may outlast the pandemic and pay off in the long run.

## Conclusions and implications

This paper provides unique insights into the situation of CABs in the COVID-19 pandemic and their response to it. The study presented builds upon international survey data from Germany, the UK, Italy, and New Zealand and provides empirical data on their economic and operational situation as well as their impact on digitalization in this core sector or the functioning of the economy.

The descriptive results of our study show how severely CABs were affected by the challenges posed by the pandemic. Across all countries surveyed, a general decline in orders was observed among accredited CABs, both domestically and internationally. New Zealand was the least affected, followed by Italy, while German CABs faced the most substantial decline. Against the backdrop of the dependence of German CABs on the heavily disrupted manufacturing sector, the CABs associated with this industry were most negatively affected by the pandemic. The time at which the survey was carried out in the respective countries also proved to determine perceptions and figures. Furthermore, differences across CA activities were observable on average, e.g., medical laboratories’ rise in order demand as opposed to inspection bodies most heavily faced with restrictions and accordant decline. Therefore, CABs offering essential services were not as heavily affected as those offering non-essential or services for highly disrupted sectors. Essentially, the severity of the pandemic’s impact is determined by differences in the sectors served by CABs. Our study thus extends the initial findings from the CA sector, e.g., Summers and Charrington [18], who drew similar conclusions.

In the COVID-19 pandemic, studies have shown the importance of digital capabilities and resources [65, 67]. Digital capabilities and resources ensure the continuity of processes and services and safeguard interactions among employees and external stakeholders [70]. Our study found differences between participating countries. For example, CABs from the UK and New Zealand prove more digitalized and thus adapted more easily to the newly emerging needs. In contrast, German CABs were significantly less digitalized before the pandemic, and the personnel was not adapted to the digital environment. The crisis exposed an overreliance by CABs on traditional approaches (e.g., on-site auditing and inspections), which were severely impacted during the pandemic. This reliance (and absence of remote solutions) made it difficult to deliver their services. The pandemic did indeed accelerate the uptake of digitalization of the CA sector, as our data shows, but it also revealed significant variations across countries in our sample.

Future studies should continue the direction of the research established in this paper. First, the emergency preparedness and vulnerability of the CA sector need further scrutiny. Future studies should address important questions related to quality infrastructure (i.e., how should CA activities be coordinated in times of crises? How can remote services enhance local economies and/or their innovation systems?) as well as important questions related to strategic positioning and operational matters of individual CABs (i.e., what service portfolio is optimal for a CAB to minimize its risks?). Second, the digitalization of CA is going to continue. More studies are needed to support this effort. For example, future studies should develop a digitalization index to establish the level of digitalization across the sector. Simultaneously, future studies also need to describe the pathways for digitalization, which would ensure the continuity of CA while also enabling the digitalization of the sector. In both of these areas, particular attention needs to be paid to differences between the subsets of CA services (i.e., differences between inspection bodies and certification bodies). Finally, it would be worthwhile to revisit existing standards, CA procedures, and trade agreements and investigate whether and in what ways the current system might affect digitalization progress.

Our study has implications for practice. Exposing CABs’ vulnerability in this crisis will allow policymakers and industries to draw conclusions for the post-pandemic world, which is likely to depend even more than before on a functioning CA system. Governments in all countries have reacted quickly with measures to ensure the survival of the organizations. CABs also showed substantial resilience in the face of the pandemic and continued to offer vital services in these difficult times. Nevertheless, their focus in the post-pandemic world should shift to structural and infrastructural measures to improve their digital capabilities [67].

Accreditation services, as well as services provided by CABs, must become aligned with the needs of the new digital environment. For example, all actors involved in the quality infrastructure need to develop skills and services to extend their scope and include the provision of remote services. Likewise, further investments need to be channeled into using a broad spectrum of technologies, such as remote sensing, robots, and other technologies [19, 71]. These trends are already observable in the sector providing CA services. Not only CABs themselves but also accreditation bodies have switched to remote procedures with the onset of the pandemic enabling CABs to seek accreditation despite facing restrictions associated with the pandemic [72].

However, there are a few limitations to our study. First, our study relies on self-reported data from participants. More objective data can be used in future studies. However, we intended to assess the situation rapidly during the pandemic and rely on participants’ perceptions of the situation. Second, we were dependent on national accreditation bodies acting to distribute the survey instrument, and response rates in the four countries differed and could not fully guarantee the representativeness of the data. At the same time, our study aims to highlight the challenges during the pandemic and compare them across countries in order to draw lessons from the pandemic. Our aim was not to precisely determine the impact of the pandemic in the individual countries. Despite these limitations, our study provides important insights into the changing nature and struggles of CABs during the pandemic and paves the way for future research in this area.

## Data Availability

The survey data are publicly available as a report at www.qi-fokus.de

## Footnotes

1 The *p* values refer to a variable’s significance level. *P* values below 0.01, 0.05, and 0.1 indicate statistical significance, respectively.

## Code availability

The execution files used to calculate the statistics are available on request from the corresponding author.
